# Evolutionary biophysics in Brazil

**DOI:** 10.1007/s12551-026-01456-5

**Published:** 2026-09-12

**Authors:** Lucas Bleicher

**Affiliations:** Departamento de Bioquímica e Imunologia, Instituto de Ciências Biológicas, Universidade Federal de Minas Gerais, Av. Antonio Carlos, 6627, Pampulha, Belo Horizonte, MG 31270-901 Brazil

**Keywords:** Evolution, Speciation, Evolutionary biophysics, History of science

## Abstract

Physicists have been fascinated by the biological sciences for many years, but it has also deemed not amenable to be studied by the molecular sciences—for most of recorded history it was actually believed that biology had its own laws, a view that started to erode when the synthesis of urea on a laboratory posed a question to the vital principle. In the twentieth century, many physicists ventured into biology in general and evolution in particular. Here, we present a panel of evolutionary biophysics in Brazil, based on literature review, investigation of scientific meeting annals and personal communication with key names in Brazilian academia who traversed the barriers between physics and evolution. The review focuses on scientists from Brazilian institutions, including foreigners that moved to Brazil. Most of the research groups discussed here originated from a physicist’s interest in biology, but the opposite was also possible. Most of the scientists presented in this review are still active in the area, or advised PhDs that created new groups of evolutionary biophysics themselves. Organizational initiatives are also briefly discussed. This review intends to not only be a “who is who” in Brazilian evolutionary physics, but also share a few historic events that brought scientists to this area.

## Introduction

This commemorative issue of *Biophysical Reviews* celebrates the 90th birthday of the Brazilian Biophysical Society in 2026. The Brazilian Evolutionary Biology Society, though, was founded only in 2024. This is not because evolution is a recent research area in Brazil, but because its scientists have been scattered through many scientific societies and their respective meetings, such as the Brazilian Society of Genetics, several societies on biological sciences and, on occasion, the Brazilian Physical Society.

For a long time, biology had been considered detached from other sciences. *Vitalism*, the idea supporting that life could not be explained by physical or chemical principles, remained popular even well after German chemist Friedrich Wöhler synthesised urea in the laboratory, without the necessity of a “vital force”. Still, Erwin Schrödinger, in his seminal book *What is Life*, wrote already in its first chapter, that present-day (in that case, the early twentieth century) physics and chemistry was obviously unable “to account for the events in space and time that take place inside a living cell” (Schrödinger 1944). It is then followed by a discussion on Statistical Physics. It is no coincidence that, while investigating the history of research groups involving physics and evolution in Brazil, many of the findings were on the Statistical Physics sessions of the National Meeting on Condensed Matter Physics (since 2018 branded *Autumn Meeting*). Despite the name, it also hosted sessions on most areas of Physics, while Nuclear Physics and Particles and Fields had exclusive meetings, later united as the *Spring Meeting*. As Thadeu Penna, one of the pioneers in the field in Brazil (discussed in the section The UFF group) mentioned in a personal communication for this review, biological and social systems attracted physicists from condensed matter because *even though the human population is on the order of 10*^*9*^*, that would still be far less than the atoms in a gram of salt, making it much easier to simulate on a computer*. Most of the past and present contributions to evolutionary biophysics in Brazil discussed in this review came from physicists (or other scientists with a background in molecular sciences) who discovered biological problems that could be investigated using frameworks they already were familiar with. The opposite path—starting from a biological background in evolution and then looking for a physical approach to solve a particular problem—was also possible, though, and it is illustrated with the interesting case of a group of scientists nucleating in Bahia. Also, one of the most relevant scientists in the field was neither a biologist nor a physicist but devoted most of his career to biological evolution through a physical framework, and will be discussed in the next section.

**Fig. 1:**
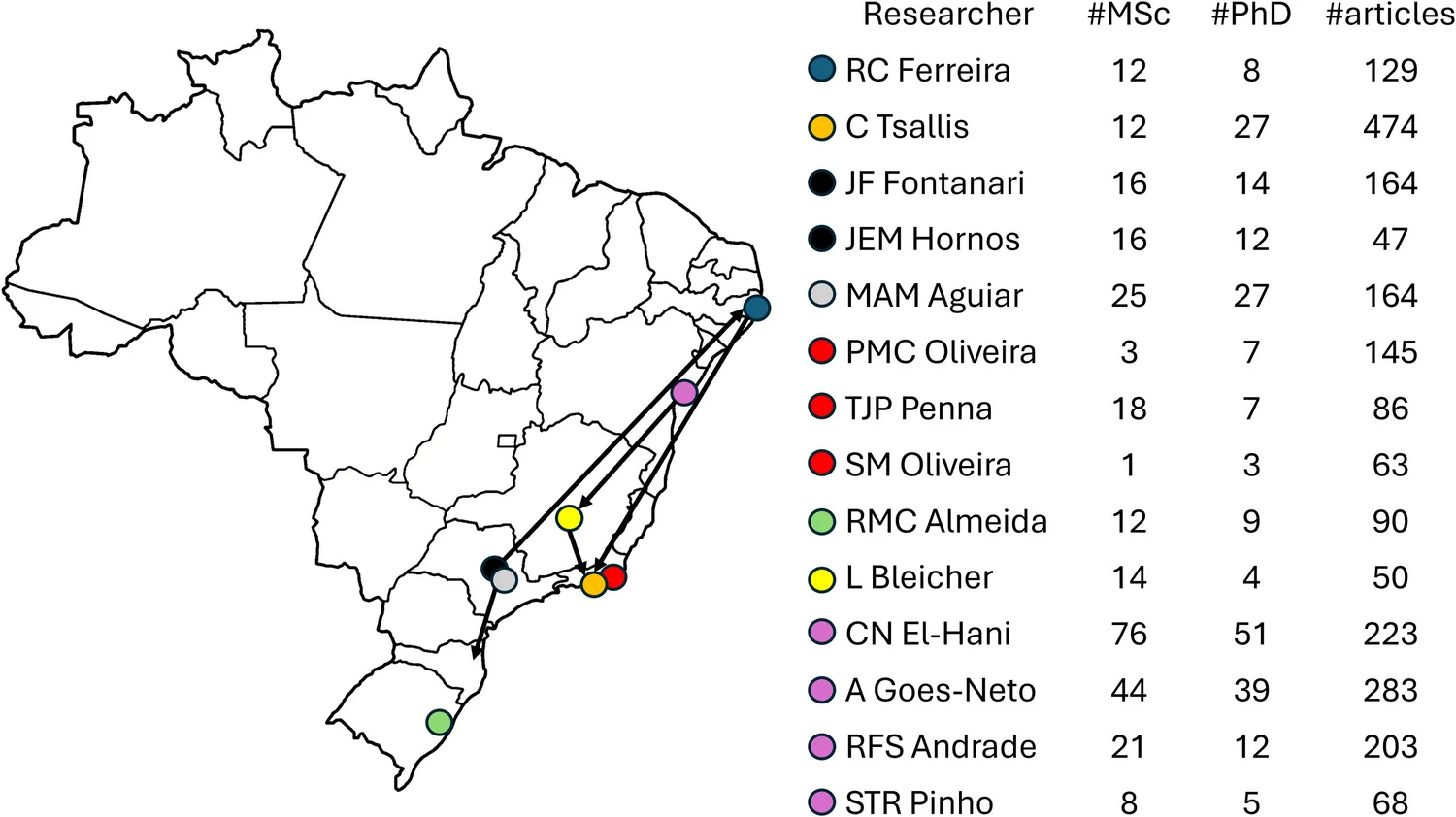
Geographic distribution of the main groups discussed in this review. Blue: Recife (PE). Orange: Rio de Janeiro (RJ). Black: São Carlos (SP). Grey: Campinas (SP). Red: Niteroi (RJ). Green: Porto Alegre (RS). Yellow: Belo Horizonte (MG). Purple: Salvador (BA). Arrows represent movement of group members and Phd students that started their own groups in other cities

This review, based not only on the published work of scientists in Brazil but also on personal communication with members of the community and on abstracts and scientific programs of past scientific meetings, aims to present a portrait of different research groups (most still active) working on evolutionary biophysics in Brazil (Fig. 1)—from the application of statistical physics methods to pre-biotic evolution to alternatives for phylogenetic reconstruction.

## Early history—Ricardo Ferreira and Constantino Tsallis

The earliest examples we could find on the interface between the physical sciences and evolution in Brazil came from two notable names in Brazilian Physics. Ricardo de Carvalho Ferreira, whose publishing record starting from the 1940s, graduated in Chemistry and worked in several institutions, including a period at Caltech while Linus Pauling was the chair of the Department of Chemistry. Many of his early works were focused in the application of the quantum mechanical descriptions of chemical binding. This included the first quantum chemistry calculation entirely developed in Brazil, using a UNIVAC 1105 from the Brazilian government agency responsible for the census, IBGE.

Already in 1953, a short communication to Nature, *Resolution of Racemic Mixtures by Symmetrical Agents* (Ferreira 1953), contained at its end a foreshadowing of his future interests:

Finally, we wish to stress the implications of these findings (as well as those of Powell) on the problem of the origin of molecular dissymmetry in living matter. Processes such as those recently described are probably more likely to have occurred in the past than the activation of reactive racemic mixtures by naturally polarized light, even if they lead to a spontaneous decrease in the entropy of the system.

It was only in the 1970 s, however, that Ferreira would give further attention to biological problems, especially the physical chemistry of enzymes. The focus on evolution would happen in the next decade, starting with a collaboration with one of the most renowned physicists in Brazil—Constantino Tsallis. Born in Greece, Tsallis emigrated at four, and after studying Physics in Argentina and then getting his PhD in France, he moved to Brazil and started a long career at the Centro Brasileiro de Pesquisas Físicas (CBPF), notorious for his generalization of the Boltzmann–Gibbs statistics, currently at nearly 13 thousand citations (Tsallis 1988).

In 1983, Tsallis and Ferreira teamed up to publish *On the origin of self-replicating information-containing polymers from oligomeric mixtures* (Tsallis and Ferreira 1983), on which pre-biotic evolution is investigated using the renormalization group formalism for condensation reactions such as those that polymerize DNA, RNA and proteins, proposing that the transition to an information-containing self-replicating polymer is a critical phenomenon. A year later, with his student Saul Jacchieri, he published *Why are there two Kinds of Chains in Tetrameric Hemoglobins? (*Ferreira and Jacchieri 1984*)*, suggesting that the ɑ_2_β_2_ tetramer gave an evolutionary advantage due to an enhancement of the mean allosteric free energy between 10 and 35 °C.

Other collaborations with Tsallis on pre-biotic evolution as a critical phenomenon followed (Ferreira and Tsallis 1985; Tsallis and Ferreira 1986), as well as other studies on the self-replication of biological polymers (Ferreira 1987; Ferreira and Coutinho 1993), consolidating the intersection between the molecular sciences and evolutionary biology as Ferreira’s definitive area of research.

In the 1990 s, Ricardo Ferreira worked on the evolutionary origin of the genetic code, from *Why 4(3) codons?* (Ferreira 1995) to the chirality of DNA and proteins (Ferreira 2002; Soares et al. 1997).

In 2000, in *On the classes of aminoacyl-tRNA synthetases and the error minimization in the genetic code *(Cavalcanti et al. 2000), Ferreira defines two types of mutations (those that change or not the class of the involved amino acids) as a consequence of the existence of two aminoacyl-tRNA synthetases, with the mutations that do not change classes would be responsible for most of the minimization of the genetic code, supporting pre-biotic models on which duplications or modifications of existing aminoacyl-tRNA synthetases led to the addition of new amino acids.

Besides his extensive record of regular scientific articles, Ricardo Ferreira published books, chapters and magazine articles on evolution and passed away in August 2013 at 85.

While Constantino Tsallis has focused in other areas after his initial collaborations with Ferreira, he still made contributions to evolutionary physics, particularly to the Bak-Sneppen model of species co-evolution (Bak and Sneppen 1993). The Bak-Sneppen was the first physical model of evolution that supported the idea of punctuated equilibrium, on which species are stable for a good part of their history and drastic evolutionary changes occur in specific and uncommon moments.

Geographic distribution of populations was simulated by Tsallis in a model where being nomadic or sedentary are transmissible traits (Tsallis 1993), which was later improved to investigate the relations between mutation rates on polygenic variance in migrating populations (Da Silva and Tsallis 1999).

In collaboration with Francisco Tamarit and Sergio Cannas, from Universidad Nacional de Córdoba, Tsallis investigates the sensitivity to initial conditions, an aspect not previously assessed for the model. It does indeed present a power-law sensitivity to initial conditions at the self-organized critical state (Tamarit et al. 1998).

In 2008, on the occasion of Ricardo Ferreira’s 80th birthday, Constantino Tsallis published, for the Journal of the Brazilian Chemical Society, the letter *My friend Ricardo Ferreira, an impressive natural philosopher* (Tsallis 2008).

## Physics and evolution at São Paulo state

After graduating in Physics at UFRGS, José Fernando Fontanari obtained his PhD in 1988 at the São Carlos campus of the University of São Paulo. Fontanari was studying artificial neural networks, which was also his research subject during his post-doc at John Hopfield’s group at Caltech. That period resulted in a publication with Ron Meir that proposed a model to study how learning, a non-inheritable feature, would affect the evolution of asexual populations significantly in comparison to the baseline where learning is not present (Fontanari and Meir 1990).

This was followed, years later, by a solid record in various aspects of biological evolution analyzed, starting by revisiting Manfred Eigen’s quasispecies model, on which self-replicating biomolecules are described as under Darwinian selection, with the concentrations of each molecule following differential equations. In 1996, Fontanari formulated the model using a populational genetics framework, which presents similar results to the kinetics formulation but can explore the parameter space more thoroughly (Alves and Fontanari 1996). He then explored another aspect of evolution—the role of altruism. In 1999, using a model for asexual individuals that incorporated altruistic and non-altruistic behaviour, it was demonstrated that mutation and migration have a detrimental effect on altruistic populations (Silva and Fontanari 1999), and he later revisited altruism in evolution using the model from Motoo Kimura, famous for the introduction of the neutral theory of molecular evolution (Fontanari and Serva 2014). He also investigated topics such as the *Muller’s ratchet*, the process on which asexual populations accumulate deleterious mutations (Colato and Fontanari 2001), the evolution of compartmentalization (Fontanari et al. 2006), the Baldwin effect, on which natural selection could be affected by learned behaviours (Fontanari and Santos 2017) and, more recently, has been applying game theory to evolution (Mariano and Fontanari 2024).

In addition to his prolific scientific production in the area, Fontanari also advised many PhDs, some of them also very active in the interface between physics and evolution. Alexandre Colato, now at UFSCar, has a long collaboration with University of New Mexico’s Philip Gerrish, studying subjects such as a negative effect of genetic linkage (simultaneous inheritance of difference genes caused by co-localization) on natural selection (Gerrish et al. 2007), the relation between natural selection and recombination (Gerrish et al. 2020) and, very recently, on how evolution can show predictability rather than being exclusively a retrospective science (Gerrish et al. 2026).

Paulo Roberto de Araújo Campos, whose PhD thesis under Fontanari’s supervision focused on pre-biotic evolution, recently became a professor at the Federal University of Pernambuco, having worked, among other subjects, with mathematical models for evolutionary rescue, the persistence of species heading for extinction by adaptation to drastic effects (Campos 2025; Mohammadi and Campos 2025).

José Eduardo Martinho Hornos, a Fontanari colleague at IFSC, started his career studying high energy physics, group theory and symmetry, and then developed an interest in physical biology. In 1993, Hornos published *Algebraic model for the evolution of the genetic code* (Hornos and Hornos 1993), searching for a symmetry group compatible with the codon assignment to amino acids. He then devoted a good part of his career to that problem, also in collaboration with Michael Forger from the Mathematics and Statistics Institute at the São Paulo USP campus, later including phylogenetic analysis of mitochondria, which have a non-standard genetic code (Hornos et al. 2004) and expanding the symmetry analysis to gene regulation (Ramos et al. 2010).

Not far from São Carlos, another group on Evolutionary Physics appeared in Campinas in the late 2000 s, when Marcus Aguiar, who already had a solid career in complex systems since the late 1980 s, started a collaboration with Yaneer Bar-Yam and Michel Baranger during a series of visits to the MIT in Boston. The publication of *Global patterns of speciation and diversity* (De Aguiar et al. 2009), where simulations of populations with sexual reproduction, speciation and dispersal were found to reproduce biodiversity dependences observed for many species, caught the attention of many students both from Physics and Biology, and soon Aguiar was mentoring several post-graduate students on projects related to evolution and speciation.

In the following years, his group published several articles on neutral models of evolution and on the construction and analysis of phylogenies. In *Moran model as a dynamical process on networks and its implications for neutral speciation*, this allele frequencies change model is simulated as a network where nodes represent genes and links connect individuals that can mate, and shows populations on which individuals mate with neighbours only are much more affected by mutations, and that when mating also requires genetic proximity there are two opposing trends—diversity can increase due to more effective mutations or decrease due to similarity between mates. The result is the formation of reproductively isolated local groups even in the absence of selection, a phenomenon called topopatric speciation (De Aguiar and Bar-Yam 2011).

In another interesting physical modeling of the geographical effects on evolution, Aguiar’s group represented fluvial structures as branched networks with a root node corresponding to confluence and upstream nodes representing tributaries, which is then used to model the interactions between river populations. It was observed that with more branching nodes there is an increase in stability in downstream populations, and the river structure buffers against synchronised dynamics (Yeakel et al. 2014).

By associating neutral models to phylogenies, Aguiar’s group correlated their parameters (number of genes, living areas, mutation rates) to phylogenetic tree properties such as balance and speciation rates. In *Signatures of Microevolutionary Processes in Phylogenetic Patterns *(Costa et al. 2018), they used the model Aguiar and colleagues published in 2009 (De Aguiar et al. 2009), which considers the effect of genetic and spatial proximity on mating with sexual reproduction, to simulate parapatric and sympatric speciation (i.e., occurring in adjacent or in the same geographic areas, respectively) and constructed phylogenetic trees, which were compared to known cases of evolutionary radiation for animals and plants. It was found that species richness is higher with less gene flow and a large number of loci, and that genome size has a large effect on the time for species number equilibration—speciation events occurring on a reasonably constant rate for small genomes, while large genomes cause earlier equilibration and the behaviour seen in adaptive radiation, with many early speciation events and then species accumulation declining over time. Also, balanced trees with constant speciation rates are seen only in the sympatric case, and the model predictions agree with empirical data from evolutionary radiation in parapatry or sympatry (Costa et al. 2018).

In recent years, Aguiar has incorporated to his evolutionary models aspects such as mito-nuclear interactions (Princepe et al. 2022), variable geographic barriers (Princepe et al. 2024), assortative mating (Lizárraga et al. 2024), antagonistic interactions (De Godoy and De Aguiar 2026) and other factors.

Even though Marcus Aguiar’s interest in evolution happened later in his career, it already radiated from Campinas. Sabrina Borges Lino Araujo, who completed her PhD in 2010 under his supervision, is currently a professor at UFPR, studying for example the effects of landscape fragmentation on speciation (Ciccheto et al. 2024).

## The UFF group

When José Fernando Fontanari, discussed in the previous section, defended his PhD thesis on artificial neural networks, one of the committee’s members, Paulo Murilo Castro de Oliveira, found out that this kind of problem would be very interesting for the research on computational physics in his home institution, the Fluminense Federal University (UFF), in Niteroi. He brought the thesis to his PhD student Thadeu Josino Pereira Penna, they shortly after published an application of neural networks to culture and evolution (Penna and De Oliveira 1992) and, together with Suzana Moss de Oliveira, a fruitful research group on biological physics was born.

In a visit to Niteroi, German physicist Dietrich Stauffer, from the University of Cologne, discussed his interest in studying biological aging. The idea was simulating the Mutation Accumulation Theory, one of the major models in biological senescence, based on the idea that harmful mutations only having an effect at an old age, i.e., after they could affect reproduction, would accumulate because they would not be removed by natural selection, resulting in senescence. This theory was proposed by Peter Medawar, who was born in Brazil but soon moved to the United Kingdom. Thadeu Penna modeled mutation accumulation as a bit-string to represent deleterious mutations, information on when will such mutations have an effect in the lifetime of an individual, reproduction age, how many deleterious mutations an individual can suffer and still survive and effects of food and space restriction (Penna 1995). The simplicity and computational efficiency of this implementation, and also the fact it was the first model to reproduce the Gompertz-Makeham law (which states an exponential increase in mortality with age) made *Penna’s model* very popular (Stauffer 2009).

With Stauffer, Penna and Moss de Oliveira published two articles on the senescence of fish by applying their model to the Northern Cod (Moss De Oliveira et al. 1995) and the Pacific Salmon (Penna et al. 1995). The model was later explored by the group to compare sexual and asexual reproduction (Stauffer et al. 1996) and the inheritance of longevity (De Oliveira et al. 1998).

In 2001, Suzana Moss de Oliveira and her PhD student Adriano de Oliveira Sousa incorporated in Penna’s model the concept of antagonistic pleiotropy, one of the major hypothesis in biological aging, which proposes that genes giving a positive effect in early life can also cause negative effects at an old age. With such addition, the model can reproduce both the exponential increase in mortality seen in humans and decelerating mortality as seen in the fruitfly (Sousa and Moss De Oliveira 2001).

Other modifications of the Penna model by the group included adding phenotype selection to study sympatric speciation (Sá Martins et al. 2001), simulating DNA inheritance and reproducing the “mitochondrial Eve” (Deoliveira 2001), polyploidy (Sousa et al. 2003) and positive mutations (Moss De Oliveira et al. 2004).

In *The zipper effect: Why different positions along the chromosome suffer different selection pressures*, Paulo and Suzana de Oliveira used the bit-string model to analyse the effects of the relation between loci position on a chromosome and mutations (De Oliveira and Moss De Oliveira 2011). According to their model, functionally related gene clusters are more likely to be found near chromosome centers while isolated genes are more common on chromosome tips, which is compatible with what is observed for HOX genes.

Outside of the bit-string Penna Model, Paulo de Oliveira proposed a very interesting application of a physical model to evolutionary biology in 2013, applying the famous Ising model to evolutionary trees reconstruction (De Oliveira 2013). In the model, extant species are represented as particles moving along an axis and freely passing through each other, initially released on the origin with random velocities and then interacting via energies with coupling constants that include the difference between the species, and the method is then used to infer their phylogenetic tree.

Even though Paulo Castro de Oliveira passed away in 2023, Suzana Moss retired in 2013 and Thadeu Penna, who moved to the Volta Redonda campus is also contemplating retirement, a newer generation is still keeping evolutionary physics research alive at UFF—Marcio Argolo Ferreira de Menezes, a former student from Thadeu Penna who published *Why Trees Live Longer* (De Menezes et al. 1996) while still an undergraduate, has been working on subjects such as the evolution of plumage colors in birds (Oud et al. 2023) and was the PhD advisor to Nuno Crokidakis, who among many other subjects studied the effects of randomness in extinction via the Allee effect, on which population size affects fitness (Pires et al. 2022).

## Evolutionary physics in Southern Brazil

Coming from nuclear physics, Rita Maria Cunha de Almeida published on cellular structures and neural networks, then got closer to evolutionary physics in the late 90 s, having collaborated with the UFF group, discussed in the previous section, in two papers on biological aging (Almeida et al. 1998; Almeida and Moukarzel 1998), and then creating her own variations on the Penna model—specifically, by analyzing selection (Medeiros et al. 2000).

In 2008, in a work that involved gene/protein association analysis, phylogenetic analysis over multiple species for genes involved on apoptosis and those obtained from the association network and analysis of mutation databases, Almeida’s group inferred a detailed panel on the evolution of apoptosis and genome-stability in humans (Castro et al. 2008).

In a 2011 study, Dalmolin and colleagues defined an evolutionary plasticity measure, related to the changes a gene family tolerated during its evolutionary history, and analyzed groups of orthologs, i.e., homologous genes derived through speciation. It was found that plasticity correlated with the average evolutionary distance among ortholog groups, and genes associated with lethality are more common in less plastic ortholog groups (Dalmolin et al. 2011).

In another interesting application of complex network analysis to evolution Ferreira and colleagues investigated why more connected and less clustered proteins in protein–protein association networks are usually related to genes presenting more paralogs and those with conserved functions have fewer paralogs are highly connected and clustered. A model where genes in a network are created de novo or from gene duplications was simulated and found to describe different topological distributions and correctly predicted the percentages of gene duplication and de novo acquisition in order to reproduce the links and nodes observed in Eukaryotic protein networks (Ferreira et al. 2013).

Dr. Almeida was the PhD advisor of Physicist Jeferson Jacob Arenzon, who also became a professor at UFRGS since 1996, and applied game theory to study predator–prey dynamics in the 2010 s (Cazaubiel et al. 2017; Lütz et al. 2017, 2013).

In 2014, the Physics department at UFRGS hired Daniel Gamermann, who worked with particle physics and then encountered physical biology while a post-doc in Spain. In his first work as a correspondent author after returning to Brazil, Gamermann studied duplication and divergence, the most common origin of new protein functions in evolution, by modeling it using a network model and its dynamics as a Markov process on which, at each time step, a node (representing a protein) randomly duplicates and the copies can independently mutate afterwards (Sudbrack et al. 2018).

## Coevolution and structure–function studies at UFMG

In 2005, bioengineer Rama Ranganathan visited Brazil for the third edition of the Brazilian conference on Bioinformatics and Computational Biology (X-Meeting), where he presented his Statistical Coupling Analysis (SCA). This method quantifies sequence conservation and coevolution in analogy to energies in a Boltzmann distribution using the probabilities of occurrence of each residue in a position for conservation, and their occurrences in a position for an entire alignment and upon the presence of a different residue in another position (a “perturbation”) for correlation, described by the authors as the “statistical coupling” (Lockless and Ranganathan 1999). SCA was initially described as a means to detect “allosteric paths” in a protein: “statistical couplings” between the positions in a multiple sequence alignment were calculated, resulting in a matrix with “perturbations” as columns and its “statistical couplings” against all other positions as lines. Clustering the resulting matrix and removing low signal lines and columns would result in a set of residues, postulated to be the “allosteric core” of a protein. We applied the SCA method to the family of Fe/Mn superoxide dismutases (Bachega et al. 2009) with Richard Garratt’s group in the University of São Paulo campus at São Carlos and found an unexpected result—instead of a single set of residues, three coevolving sets were found on that protein family, with most residues in such sets having been previously described as determinants for iron binding, manganese binding or dimeric structure (as opposed to tetrameric as in some previously described proteins in that family). Our interpretation to that result was that, because protein families can commonly present members with different characteristics (such as metal binding, oligomeric state, catalysis and its specificities, and DNA binding), and because such characteristics are likely to be the result of several residues forming functional sites, a coevolution analysis on such a family should necessarily result in the detection of sub-family determinants, provided that the alignment is sufficiently populated by members of each sub-family. At the time with structural biologist Richard Garratt, we decided to develop a theoretical framework to explore these findings, starting with a metric that would quantify pairwise residue-specific correlation (or anticorrelation) between two positions. Having moved from Physics scientific meetings to those such as the Annual Meeting of the Brazilian Society for Biochemistry and Molecular Biology in the early 2000 s, I decided to return to the Condensed Matter Physics Meeting in 2009, the largest event of the Brazilian Physical Society, presenting the results we had at the time hoping to find interesting models that would be suitable to such problem.

That was indeed the case—a work from the group of Ney Lemke, a physicist at UNESP Botucatu, which used complex networks to study co-expression in biological cells seemed to be perfectly suited to the study of protein co-evolution: residues in a specific position (i.e., His35) would be represented by a node, while weighted edges would be present if the two positions showed coevolution in the protein family (with negative weights if the presence of a residue in one position led to the reduction of the rate of finding the other residue in the other position). Network decomposition algorithms (either trivial connected components if the coevolution network was simple or modularity maximization heuristics if it was too large) could then be used to find sets of coevolving residues. This resulted in the 2013 article *Using Amino Acid Correlation and Community Detection Algorithms to Identify Functional Determinants in Protein Families* (Bleicher et al. 2011). Later implemented as a software (Fonseca-Júnior et al. 2018), the method has been used for several applications such as rational designs of point mutants (Rios-Anjos et al. 2017), inferring the function of a previously uncharacterized protein annotated as a domain of unknown function (Coitinho et al. 2019) and discovering a novel, clade-specific DNA-recognition motif (Lima Afonso et al. 2013).

More recently, our group became interested in studying the evolution of transthyretin using methods in biophysics and theoretical chemistry. An interesting example of neo-functionalization, this thyroid hormone and retinol binding protein originated from the duplication of a gene that coded for a completely different function—the second enzyme in a uric acid degradation pathway. By conjugating ancestral sequence reconstruction, X-ray diffraction, isothermal titration calorimetry and molecular docking, we were able to re-enact this neofunctionalization event (Carrijo De Oliveira et al. 2021), showing the early substitutions after gene duplication were sufficient to the loss of the original enzymatic function and also the gain of a modern-like thyroxine binding mode. Studying the evolution of mechanisms of reaction, binding to metals and association with other proteins is currently underway using physical methods such as classical and hybrid (QM/MM) molecular dynamics, crystallography and further analysis of reconstructed ancestral sequences.

Mariana Torquato Quezado de Magalhães, who collaborated in such projects while in the Biological Sciences Institute at UFMG, is now at the Instituto Militar de Engenharia in Rio de Janeiro, also applying biophysical and molecular evolution methods to study proteins in the same uric acid degradation pathway, having recently characterized human OHCU decarboxylase, the third enzyme in the pathway, that is still expressed in humans despite the loss of the first two enzymes during the evolution of primates (Rodrigues et al. 2026).

## From biology to physics in Bahia

In most of the examples found for this review, evolutionary biophysics groups originated when physicists developed an interest in biology and evolution. A very interesting exception to that rule happened at UFBA, when biologist Charbel Nino El-Hani, who had a very long career on the history and philosophy of science which included studying the very process on which physicists decide to explore biology (Joaquim et al. 2015, 2019), together with biologist Aristoteles Goes-Neto teamed up with physicists Roberto Fernandes Silva Andrade, Flora Souza Bacelar and Suani Tavares Rubim de Pinho to start a group on evolutionary physics.

In 2010, the team published *Comparative protein analysis of the chitin metabolic pathway in extant organisms: A complex network approach* (Góes-Neto et al. 2010). While applying complex network theory and algorithms to biological systems is not new, here an application to evolution is presented. Adjacency matrices between proteins were built according to their sequence similarity, and those were used to construct complex networks where nodes correspond to proteins and edges are present when the two connected proteins present similarity above a threshold. Network analysis through community decomposition and centrality analysis allows one to infer phylogenies for very large datasets, even when mixing non-homologous proteins, in agreement with the regular methods of maximum parsimony, maximum likelihood and Bayesian inference.

This method has been furtherly refined in *Detecting Network Communities: An Application to Phylogenetic Analysis*, which found out that all bacterial phyla and, in some cases, also classes correspond to the modules detected by the analysis (Andrade et al. 2011). In a more audacious application from 2015, the evolutionary origin of mitochondria was investigated. While its bacterial origin is a scientific consensus, it is not clear what living bacteria are closest to mitochondria. Community structure analysis from gene networks went against Rickettsiales being the closest extant clade, and Alphaproteobacteria might be more likely.

One of the limitations of the complex network model for phylogenetic reconstruction was the lack of a reliability metric such as bootstrap for maximum likelihood inferences or the posterior probabilities calculated by Bayesian inference. The group then created a bootstrap-like clade robustness method using the similarity matrix as the source of pseudo-replicates (Góes-Neto et al. 2018).

Other examples of the application of network science from the group include the role of synchronization in ecological networks involving positive and negative interactions (Girón et al. 2016) and an investigation on the evolution of Apolipoprotein E using both maximum likelihood and the complex network based method they developed (Benevides et al. 2016).

In 2016, Aristóteles Góes Neto moved from Bahia to Minas Gerais, and is currently a professor at UFMG.

## Other incursions into evolutionary physics

While this review started with an example of a researcher who, coming from the physical sciences, developed a passion for evolution that lasted throughout his career, there have been of course many scientists who dedicated most of their attention to other subjects but still made significant contributions to evolutionary physics.

Ronald Dickman, professor at the Physics Department at UFMG, has a prolific record on statistical physics and thermodynamics, which among important contributions on phase transitions and criticality also includes several articles on evolution. In *Hidden role of mutations in the evolutionary process* (Soares et al. 2021), a model consisting of individuals in three categories, a wild-type and two mutants, with one of the mutants having higher fitness while the other has a higher probability of mutating from wild-type, is simulated in different regimes. With rare mutations, the less-fit mutant prevails, which is very unlikely when mutations are frequent.

Dickman has also worked on bit-string models, simulating a haploid population under competition, reproduction, mutation and selection using Monte Carlo and observing a phase transition between extinction through random drift and survival (Fehsenfeld et al. 2003). In *Population dynamics in the triplet annihilation model with a mutating reproduction rate*, Dickman’s introduction is a personal account on his (in his own words, “regrettably few”) encounters with Dietrich Stauffer, who, as previously discussed, was a strong inspiration for the UFF group. In this model, which Dickman developed in the late 80 s, particles can occupy sites on a lattice and three transitions are possible—creation (a new particle is generated on an unoccupied neighboring site), hopping (a particle attempts to move to an unoccupied neighboring site) and annihilation (sets of three particles are removed when occupying adjacent sites). For this application, Dickman introduced mutation (particles can acquire a value that is kept through its lifetime and is transmitted through the creation event and observed from the simulations the appearance of localized subpopulations with similar reproductive rates (Dickman 2021).

Antônio José da Costa Filho has been working on molecular biophysics since the 2000s, establishing himself as a reference in electron paramagnetic resonance in Brazil. In 2022, he discussed the evolution of the Golgi Reassembly and Stacking Proteins (GRASPs) by uniting ancestral sequence reconstruction (ASR) and biophysical characterization (Mendes et al. 2022). GRASPs associate with Golgins and such pairs are seen in the stacking of Golgi cisternae. Both GRASPs and Golgins have paralogs in many species, and Costa Filho’s group used ASR to infer GRASPs in four moments of their evolution, the most recent corresponding to the common Metazoan ancestor (after which the GRASP55 and GRASP65 paralogs diverged) and the oldest being previous to the divergence between Haptophyta and the SAR supergroup. Biophysical characterization of these ancestors showed that GRASPs became less resistant to temperature, chemicals and pH. Flexibility also increased over time, this being more pronounced in one of the modern paralogs, suggesting different evolutionary pressures after duplication. Characterization of the ancestral proteins binding to the golgins showed earlier ancestors had no preference between them, and later there was a preference between Golgin45 and GM130, resulting in the subfunctionalization of the paralogs for the cis or trans/medial Golgi faces.

Richard John Ward, a British biophysicist who established himself in Brazil in the mid-90s, is an expert in protein engineering, mostly through Directed Evolution. This technique, which resulted in the 2018 Nobel Prize in Chemistry, can be seen as the molecular equivalent of selective breeding—a protein is enhanced by applying cycles of diversification (usually random mutagenesis and DNA shuffling) and selection (the “survival of the fittest” natural selection is mimicked by artificially choosing those with a desirable trait for the next round of optimization). Many useful proteins can be obtained by this process—Ward has been applying it to obtain enzymes for industrial applications such as bioethanol production—but commonly the goal is just its *application*, not the basic understanding of the process. Ward, however, decided to investigate the physical basis of the acquisition of thermostability via directed evolution. One of such applications was the obtention of a mutant xylanase with a T_m_ 20 °C higher than the wild-type, with heat capacity change negatively correlating with the T_m_ increase (Ruller et al. 2008). Because the four substitutions are on the protein’s surface, the authors suggested the effect on heat capacity change could result from protein-solvent interfacial effects. Years later, they decided to study the physical basis of such (directed) evolutionary change on an atomic level. By crystallizing the improved mutant and subjecting it to molecular dynamics simulations in increasing temperatures, it was found that several factors contribute to the increase in thermostability—rigidity coming up on highly flexible sites, solvation effects (especially on charged residues) and hydrophobic contacts (Silva et al. 2017).

## Events and organizations

In 2012, the Abdus Salam International Centre for Theoretical Physics started its partner branch South American Institute for Fundamental Research (ICTP-SAIFR) in São Paulo. Since then, it has been organizing several activities such as schools, minicourses and workshops, which in past years has included topics such as quantitative biology and ecology, and, in 2019, a school on mathematical models of evolution. In 2025, ICTP-SAIFR also gave rise to the Physics of Life South American Network (PoLSAN), starting with a seminar with Nestor Caticha from USP, whose long career in statistical physics included studies on the evolution of altruism (Schonmann et al. 2013, 2011), and also José Fontanari and Rita de Almeida, discussed in previous sections. The organization also hosted activities such as a minicourse on Theoretical Ecology.

The major funding agency in Brazil, CNPq has a long standing program to support multi-center research institutes, the INCTs (Science, Technology and Innovation National Institutes). The INCT on Complex Systems, created in 2008, is formed by 31 scientists on 16 institutions in Brazil, and includes many scientists with previous or current work in evolutionary physics such as Rita Almeida, Constantino Tsallis, Nuno Crokidakis and Suani Pinho.

The Brazilian Biophysical Society had recently hosted symposia on Evolution and Biophysics, with local names such as Marcus Aguiar, Richard Ward, Laila Nahum, Lucas Bleicher and Guilherme Brand while inviting international names such as Michael Harms, a longtime advocate for the collaboration between physics and evolution and a co-author of an interesting review on the subject (Harms and Thornton 2013).

## Conclusions and perspectives

**Fig. 2:**
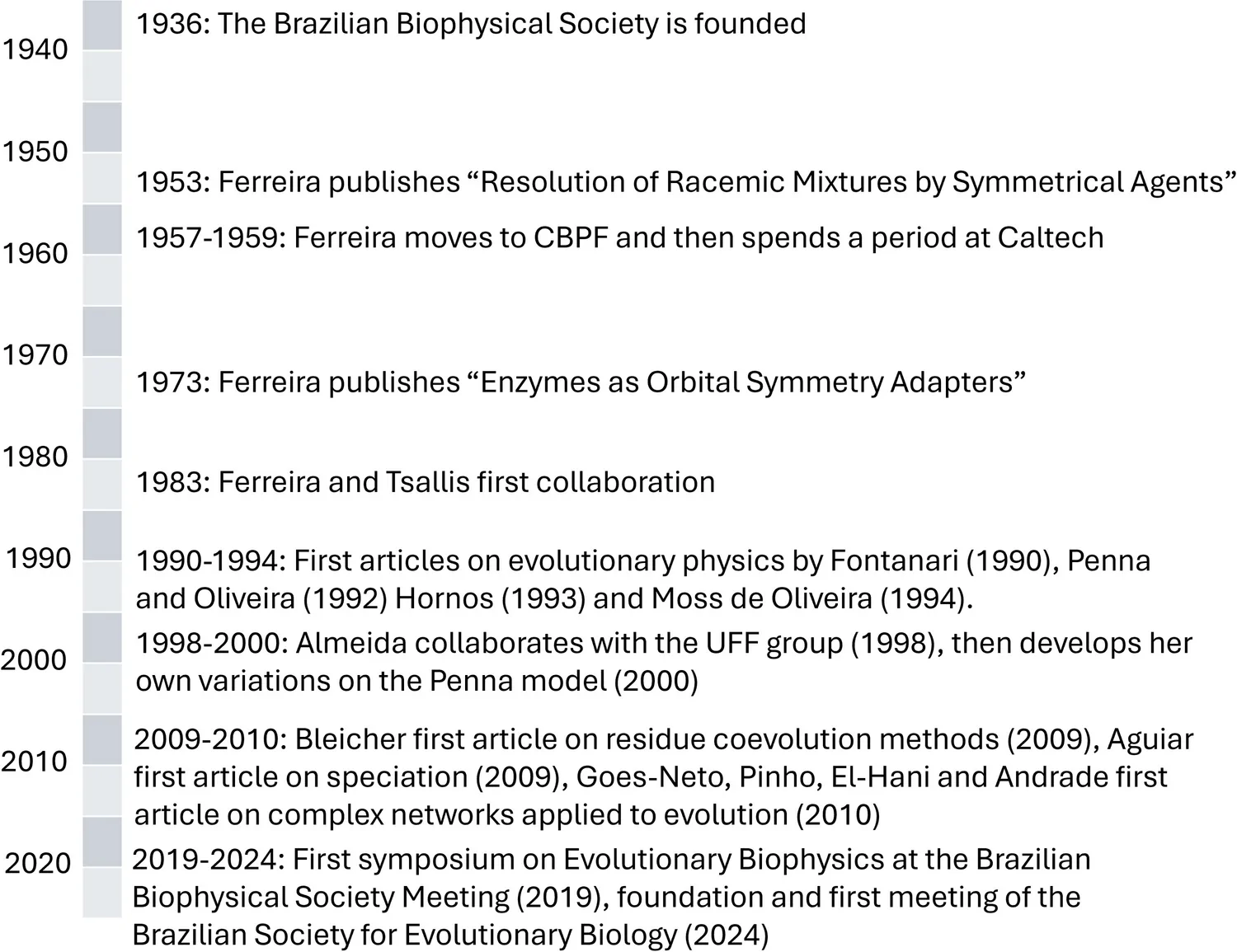
A timeline of the key events in evolutionary biophysics in Brazil from 1936 to present

Physicists have been, for a long time, fascinated with the biological sciences (and, in particular, evolution), even though, for a while, biology seemed to be “too complicated for physics”. Eventually many of them have ventured in the field, either as a passing interest or a career choice. Here, we have discussed how physics and evolution met in Brazil, either through this more common direction or the biology-to-physics path. Starting with the trajectory of Ricardo Ferreira, to our knowledge the local pioneer in dedicating a career to the amalgamation of the molecular sciences and biological evolution, several other groups nucleated in our country (a timeline of key events is shown in Fig. 2). Because Evolutionary Biophysics is a very broad interdisciplinary subject, scientists in Brazil explored many different varieties of it. Studying evolution through a Statistical Physics framework was the most common approach, with most cases involving scientists that were already active in other problems and then creating or adapting models to biological evolution. The scientists using such approaches mentioned in this review, while scattered in Brazil, are all in Physics departments. Coevolution at the residue level is discussed by evolutionary biologists at least since the seventies, but caught the attention of physicists in the 2000 s when Rama Ranganathan presented it with an inspiration from Boltzmann’s statistical mechanics, leading to applications and new developments in Brazil. Because of its applications to molecular biology and protein characterization, those using such methods are mostly on biology-focused departments/institutes, as is also the case for molecular evolutionary biophysics and directed evolution, which have clear applications to protein engineering. While this suggests that even an interdisciplinary subject can become isolated, the Bahia group, which involved the formation of a team with biologists and physics to apply complex networks to an evolutionary problem, and the Unicamp group, with constant collaboration between the Physics and Biology institutes, show that evolutionary biophysics can indeed cross departmental boundaries.

The evolution of physics and evolution happened while methodological advances for both sciences (modern computers making possible the simulation of much more complex models than the early attempts to simulate evolution, the genome era allowing us to accurately reconstruct the evolutionary history of numerous species and protein families, modern phylogenetic reconstruction methods allowing reliable inference of trees with large number of genes or proteins) meant we can now tackle what was previously considered *too complicated*. We hope this review contributes to a wider recognition of evolutionary biophysics in Brazil and to foster the collaboration between existing and, hopefully, nascent research groups.

## Data Availability

No datasets were generated or analysed during the current study.
